# Evaluation of uro-oncological surgical treatment during the Sars-CoV-2 pandemic in a Brazilian tertiary oncology institution, the new world epicenter

**DOI:** 10.1590/S1677-5538.IBJU.2020.0479

**Published:** 2021-02-03

**Authors:** Gabriel Carvalho dos Anjos Silva, Daniel Kanda Abe, Rubens Pedrenho, Rafael Nascimento Vilares, Mauricio Dener Cordeiro, Rafael Ferreira Coelho, William Carlos Nahas

**Affiliations:** 1 Instituto do Câncer de São Paulo - ICESP São Paulo SP Brasil Instituto do Câncer de São Paulo - ICESP, São Paulo, SP, Brasil.; 2 Universidade de São Paulo Faculdade de Medicina Divisão de Urologia São Paulo SP Brasil Divisão de Urologia, Faculdade de Medicina, Universidade de São Paulo, SP, Brasil.

**Keywords:** COVID-19 [Supplementary Concept], Urologic Neoplasms, Oncology Service, Hospital

## Abstract

**Introduction:**

The rapid spread of coronavirus disease 2019 (COVID-19) has dramatic effects on individuals and health care systems. In our institute, a tertiary oncologic public hospital with high surgical volume, we prioritize maintaining cancer treatment as well as possible. The aim of this study is to evaluate if uro-oncological surgeries at pandemic are safe.

**Materials and Methods:**

We evaluated patients who underwent uro-oncological procedures. Epidemiological data, information on COVID-19 infection related to surgery and clinical characteristics of non-survival operative patients with COVID-19 infections were analyzed.

**Results:**

From 213 patients analyzed, Covid-19 symptoms were noticed in 8 patients at preoperative process or at hospital admission postponing operation; 161 patients were submitted to elective surgery and 44 to emergency surgery. From patients submitted to elective surgeries, we had 1 patient with laboratory confirmation of COVID-19 (0,6%), with mild symptoms and quick discharge. From the urgencies group, we had 6(13%) patients tested positive; 5 were taken to ICU with 4 deaths.

**Conclusion:**

Elective uro-oncological procedures at the COVID-19 epidemic period in a COVID-19-free Institute are safe, and patients who need urgent procedures, with a long period of hospitalization, need special care to avoid COVID-19 infection and its outcomes.

## INTRODUCTION

The rapid spread of coronavirus disease 2019 (COVID-19), caused by a novel betacoronavirus known as severe acute respiratory syndrome coronavirus-2 (SARS-CoV-2), throughout the World, has dramatic effects on individuals and health care systems far beyond those infected with SARS-CoV-2 (1). The heavy demand for resources, exacerbated by limited health system capacity, means that health care systems have become quickly overwhelmed and hospitals have become sources for virus transmission. In response, professional bodies have recommended reprioritizing surgical cases (2, 3).

Cancer patients are considered immunocom-promised due to the nature of their disease and to the treatment they are submitted (chemotherapy, radio-therapy, or surgery). Owing to more advanced age, the impossibility of receiving adequate medical care, and the fact that cancer patients have 3.5 folds risk of developing COVID-19 related serious events, all elective surgeries, chemotherapy, and radiotherapy procedures in stable patients should be deferred (4–6). The decision of postponing cancer surgeries or not will also depend on patient condition/decision and hospital capacity in providing intensive care units, ventilators and transfusion support among other resources needed for major cancer surgeries (7).

In this context, virtually all urology centers have been forced to prioritize surgical interventions for cancer patients by applying a series of restrictions on elective approaches to optimize health care resources and minimize the risk of hospital-acquired infection (2, 7–10).

According to the updated Brazilian statistics released on May 27^th^, 2020, 411.821 people had been with COVID-19, with 25.598 deaths. Our country is the new epidemic epicenter in the World. The State of São Paulo is the epicenter in our country, with 89.483 cases and 6.712 deaths, with numbers rising exponentially. The dramatic spread of this healthy crisis is leading to major changes in the management of patients with cancer, including those affected by genitourinary malignancies (10, 11).

In the middle of March our hospital, the largest in Latin America, initiated a war operation to receive patients with COVID-19 from all over the state (12). The Central Institute of Hospital das Clinicas at the University of São Paulo Medical School, a 900-bed institute, was evacuated to provide care specifically for COVID-19 patients.

In our complex's oncology center, a public tertiary hospital with high surgical volume, we prioritize maintaining access to cancer treatment as much as possible. Every patient with suspected coronavirus infection is transferred to our exclusive COVID care institute, in order to protect other patients from acquiring the infection in our institute, while maintaining access to life-saving interventions.

### Planning for Uro-oncological care

Since the index case in Brazil on February 26^th^ up to 18^th^ May we performed 205 uro-oncological procedures, including elective and urgency surgeries. On March 20th we started contingency planning, reducing the number of elective surgeries drastically. Every patient with scheduled surgery was previously screened for COVID symptoms. This was done remotely, via telephone call. Symptomatic patients had their procedure delayed, received orientation on when to seek medical attention for COVID disease and were reevaluated, by telephone call, after 14 days. Patients remotely cleared for surgery were also screened in person upon hospital admission. If symptomatic, they were evaluated by the hospital's infection control team, which then decided on authorizing the procedure or not.

Most of follow-up consultations were made by telephone. Requests for in person reevaluation were made at the physician's discretion. For every high-risk situation, placement and removal of PPE should be done according to the control of infectious diseases CDC and other guidelines (13). All patients with suspected COVID-19 or positive in any area of the institute (ER, ICU, Infirmary, Consultations) are transferred to the COVID-19 Care Institute as soon as possible.

Our team decided to recommend surgeries in patients with high oncologic risk. We prioritized patients with bladder cancer ≥T2 or untreated tumors. Re-TURBs were primarily postponed. For renal neoplasms, all patients with T2+were referred to the procedure, due to their high risk of oncological progression, in addition to radical orchiectomies for testicular neoplasia and prostatectomy for high-risk prostatic cancer. We kept performing urgent procedures, that in the oncologic institute are mainly drainage of the urinary tract due to malignant obstruction and treatment of severe hematuria.

In this study, we aim to present the experience of a tertiary oncologic institute with uro-oncologic procedures during the pandemic, demonstrating if it is safe to perform oncological surgeries during this period at a COVID- free center.

## MATERIAL AND METHODS

### Study design and participants

This is a retrospective study which was done at Instituto do Cancer do Estado de São Paulo, in São Paulo city, the most serious area of COVID-19 epidemic in Brazil until this moment, the new World epicenter. We retrospectively reviewed patients who had undergone urologic procedures due to neoplasm since the index case of COVID-19 in Brazil (26th February 2020) until 18th May 2020.

Patients developed symptoms after operation and were diagnosed with COVID-19 according to WHO interim guidance (4, 14). Laboratory confirmation of SAR-CoV-2 was done by quantitative RT-PCR on samples from the respiratory tract (4, 15, 16) which was performed by the hospital infection control team.

This study was reviewed and approved by the Medical Ethical Committee of our institute. The clinical outcomes of these operative patients were monitored up to May 27^th^, 2020, the final date of follow-up.

A descriptive statistical analysis was done. The selection criteria were patients with oncologic diagnostic, older than 18 years with urologic procedure during the COVID-19 pandemic.

### Data collection

We reviewed clinical records, nursing records, laboratory findings, and chest computed tomographic (CT) scans for all operative patients. The admission date of these patients was from February to May 17th. Epidemiological, clinical, laboratory and radiological characteristics and treatment and outcomes data were obtained with data collection forms from electronic medical records. Information included demographic, exposure history, underlying comorbidities, chest CT image, surgical type, surgical time, signs and symptoms, time from surgery to first symptoms, COVID-19 onset and ICU admission were also recorded.

### Statistical Analysis

Continuous variables were presented as median. Categorical variables were expressed as frequencies and percentages.

## RESULTS

One hundred sixty-nine patients were selected for surgery. Of these, 8 patients showed signs or symptoms of COVID-19 in the preoperative evaluation or at hospital admission. This screening was done through directed clinical evaluation. The symptomatic patients were oriented to isolation for 14 days and were reevaluated after, or if they presented any symptom of severity, they were referred to the specific service for handling COVID. All remaining 161 underwent a surgical procedure.

In addition to these, 44 patients who came to the emergency room with an urologic urgent medical condition underwent surgery during this period. None of them showed suggestive signs or symptoms of coronavirus disease at the entrance. Of the 205 operated, 17 patients presented signs or symptoms suggestive of COVID-19 infection at some point after the procedure, 7 of which had the diagnosis confirmed by quantitative RT-PCR on samples from the respiratory tract.

### Patients that did not perform procedure due to signs or symptoms of COVID-19

We had 8 patients with suspicions barred before surgery: 3 admitted to hospital with symptoms (2 fever+headache, 1 with dyspnea); 1 asymptomatic patient with altered CT image, despite having been previously cleared for surgery by the hospital infection control team; 2 COVID-like symptoms in the preoperative evaluation with an anesthesiologist. All subjects were oriented to return after 3 weeks for reevaluation; 2 2 of which informed COVID-19 confirmation by telephone call.

### Elective surgery

We had 161 patients submitted to oncologic urological surgery since the beginning of COVID-19 in Brazil. The age range was 18 to 88 years, and the median age was 66 years. 33 (20%) patients were women. There were 92 (57%) with hypertension, 34 (21%) diabetes, 21 (13 %) cardiovascular disease and 17 (10%) with chronic obstructive pulmonary disease. The ECOG was 0 for 53 patients, 1 for 75 patients. 27, 5 and 1 patients were ECOG 2, 3 and 4, respectively. 113 patients (70%) undergoing surgery had localized cancer and 48 patients with metastatic disease or a locally advanced disease with no curative treatment proposal. The median hospital stay was 3 days, ranging from 1 to 60 (Table-1).

**Table 1:** Baseline characteristics of operative patients at the COVID-19 pandemic period.

		No. (%)		
		Total (N=205)	Elective surgery(N= 161)	Urgency(N= 44)
Median Age, yr, range		66 (18-88)	66 (18-88)	65 (25-86)
Male /Female, %		155 (76%)/ 50(24%)	128 (80%)/ 33(20%)	27 (62%)/ 17(38%)
**Comorbidities n,** %				
	CVD	32 (15%)	21 (13%)	11 (25%)
	Hypertension	115 (56%)	92 (57%)	23 (52%)
	Diabetes	45 (22%)	34 (21%)	11 (25%)
	COPD	22 (11%)	17 (10%)	5 (11%)
**ECOG n,** %				
	0	59(%)	53(%)	6(%)
	1	87(%)	75(%)	12(%)
	2	45(%)	27(%)	18(%)
	3	12(%)	5(%)	7(%)
	4	2(%)	1(%)	1(%)
Average days of hospitalization, days		8	3	17
COVID – 19 positive cases n, %		7 (3.4%)	1 (0.6%)	6(13.6%)
Deaths due to COVID-19 n,		4	0	4

**CVD** = cardiovascular disease; **COPD** = chronic obstructive pulmonary disease; **ECOG** = Eastern Cooperative Oncology Group Performance Status; **COVID-19** = 2019 novel coronavirus disease.

We performed 16 laparoscopic radical pros-tatectomies, 12 open radical prostatectomies, 7 open cystectomies,1 laparoscopic partial cystectomy, 5 penectomies, 1 retroperitoneal lymphadenectomy, 1 inguinal video-assisted lymphadenectomy, 1 open pelvic lymphadenectomy, 7 open radical nephrectomy, 18 laparoscopic radical nephrectomy, 3 laparoscopic partial nephrectomy, 8 orchiectomies,1 laparoscopic nephroureterectomy, 1 open nephroureterectomy, 1 open adrenalectomy,1 laparoscopic distal urethrectomy, 48 transurethral resection of the bladder, 27 drainage of the uri-nary tract due to malignant obstruction (double j catheter implantation, transurethral resection of the prostate, nephrostomy).

Seven patients presented with some COVID-19 sign or symptom days after the procedure. Just one patient had laboratorial confirmation of COVID-19 (0.6% from all elective procedures). All the others were excluded for coronavirus infection, as described: 3 with cough and coryza were tested negative and 1 tested positive for H1N1; 1 pulmonary thromboembolism; 1 with previous dialytic renal failure admitted at ER with respiratory failure was diagnosed with hypervolemia, with improvement after hemodialysis; 1 with high clinical-radiologic suspicious without laboratory confirmation, despite repeated testing.

The positive case was a 58-year-old woman submitted to a Laparoscopic Radical Nephrectomy due to T3 kidney cancer. She developed dyspnea and desaturation a few hours after surgery, and received a diagnosis of COVID-19 infection after hospital infection control team evaluation and a confirmatory test. After six days she was discharged, with no need for intensive care unit support. The patient started with symptoms 24 hours after hospital admission. We believe that contamination was community-acquired, not associated with hospital exposure.

### Urgent procedures

Regarding urgencies, we performed 44 procedures. 32 (72%) due to urinary malignant obstruction, 2 (5%) reoperations, 10 (23%) for hematuria treatment (transurethral resection of the bladder and prostate, clot evacuation).

The age range was 25 to 86 years, and the median age was 65 years. 17 (38%) patients were women. Hypertension was present in 23 (52%) patients, diabetes in 11 (25%), 5 (11%) and 11 (25%) had chronic obstructive pulmonary disease and cardiovascular disease retrospectively (Table-1). Ten patients submitted to surgery had localized cancer and 34 patients had metastatic disease or locally advanced disease without a curative proposal. The ECOG was 0 for 6 patients, 1 for 12 patients. 18, 7 and 1 patients were ECOG 2, 3 and 4, respectively. The average hospital stay was 17 days, ranging from 1 to 65 (Table-1).

We observed 10 patients with postoperative signs or symptoms of COVID-19 infection, as listed: 6 patients COVID-19 positive (13% from all urgencies), 1 with ARDS with negative laboratory findings for COVID-19; 2 pulmonary thromboembolism; 1 respiratory distress due to urinary septic shock.

Among those infected, 5 were taken to ICU, with 4 deaths. One had epidemiological history for COVID-19. The others did not have an epidemiological factor. The average time between the date of arrival at the hospital and the onset of symptoms was 14 days (9 to19 days) (Table-2).

**Table 2:** Clinical characteristics of non-survival operative patients with COVID-19 infections.

Date of admission		Patient 1	Patient 2	Patient 3	Patient 4
		Mar 17	Apr 15	Apr 10	Apr 24
Age, years		86	80	73	77
Sex, Female- male		Male	Male	Female	Male
Epidemiological history		yes	no	no	no
**Comorbidities**					
	CVD	yes	yes	yes	Yes
	Hypertension	yes	yes	yes	Yes
	Diabetes	no	yes	yes	No
	COPD	yes	yes	no	no
	ECOG n, %	2	3	1	1
**Date of surgery**		**Mar 24**	**Apr 16**	**Apr 17**	**Apr 30**
Surgical type(elective/ urgency)		urgency	urgency	urgency	Urgency
Days of Surgical Hospitalization		21	12	35	19
Onset of symptoms		Apr 03	Apr 23	Apr 19	May 06
Date of death		Apr 09	Apr 27	Apr 27	May 13

**CVD** = cardiovascular disease; **COPD** = chronic obstructive pulmonary disease; **ECOG** = Eastern Cooperative Oncology Group Performance Status; **COVID-19** = 2019 novel coronavirus disease.

### Characteristics of non-survival operative patients

The age range was 73 to 86 years, and the median age was 78 years; 1 (25%) patient was a woman. All patients had at least 2 comorbidi-ties. There were 4 (100%) with hypertension, 2 (50%) diabetes, 4 (100%) cardiovascular disease and 2 (50%) with chronic obstructive pulmonary disease The ECOG was 1 for 2 patients, 2 and 3 for each other (Table-2).

In the present study, patients who had an unfavorable outcome were submitted to urgent procedures. All of them were admitted by the ER, with an average of 22 days of surgical hospitalization (Table-2).

## DISCUSSION

Surgery cannot be considered ‘elective’ for many patients with cancer (17). Disruption of the full spectrum of medical cancer care services will undoubtedly have a large effect on cancer-related mortality. A 5-10% decrease in survival in high-income countries has been predicted, which will account for hundreds of thousands of excess deaths, dwarfing those caused by COVID-19 -but we are missing precise data on mortality that can be used to anticipate future cancer care needs (18). It appears from the results of this study that delivering elective uro-oncological surgery during the COVID-19 pandemic is safe with minimal postoperative complications related to the virus.

Our elective surgical volume was about 120 surgeries per month. After the contingency plan, initiated on 21^st^ March, we started to perform about 50 procedures monthly. Due to the lack of diagnostic tests for COVID-19 in the Brazilian public health system since the beginning of the pandemic, we need to ration this resource as much as possible. All patients had standard pre-COVID-19 pre-operative assessments with no COVID-19 swabs taken preceding surgery (19). Screening for COVID-19 symptoms was applied at the hospital admission, and patients were excluded if any symptoms were present. During the postoperative period, all patients recovered on designated “COVID-19 negative” infirmaries or Intensive care units. From all the 161 elective cases operated until now, just 1 person presented with COVID-19 infection, initiating on the same day of the surgery, suggesting that the person was an asymptomatic infected person previously to admission, with no complication due to the infection and quick discharge.

We observed in our data survey that patients with need for urgent surgery at our institution had a higher risk of infection, with a higher rate of an unexpected outcome. This probably happened because these patients stayed in the hospital for a longer period, having visited several sectors of the hospital (ER, wards, surgical center, and many of them ICUs), thus significantly increasing exposure and risk of infection by the virus. Based on the above, we must pay attention to low-risk cancer patients who are postponing surgeries. Depending on how long the quarantine lasts and the contingency plan for hospitals, these patients may need urgent surgery, increasing their risk of infection and death by COVID-19 in addition to the possibility of tumor upstaging and changing the oncological prognosis.

Our study supports the continued delivery of appropriately triaged uro-oncological surgery during the COVID-19 pandemic. From now, with more COVID-19 tests available, we started to admit the patients 2 days before the surgery, performing RT-PCR and computed tomography of the chest. A thorough assessment accounting for the risks and benefits for each case is necessary (19), and as the duration of the pandemic progresses, the ability to continue delivering oncological care will greatly help mitigate the longer-term impact on delaying cancer treatment of non-COVID-19 patients. However, we need to enforce precaution to patients who need urgent intervention, to avoid the increased risk of contagion, as assessed here.

The disease is changing medical practice and the way of life throughout the World (8, 20). In the time of crisis, uro-oncological surgeries should be centralized in tertiary urological centers that should ideally remain COVID-19-free sanctuaries, to guarantee high-quality, timely, and safe treatments to oncological patients. The health care fallout of the COVID-19 pandemic will surely be measured by the direct morbidity/mortality of the virus as well as the secondary effect that will include significant harm from the delay of oncological therapies, including surgeries (21).

## CONCLUSIONS

Patients who underwent elective uro-oncological surgeries at our institution during the pandemic period, in the epicenter of COVID-19 in Brazil, while new cases and deaths are in dizzying ascension, presented low, and therefore safe hospital exposure to COVID-19 infection. Patients submitted to urgent procedures, with a higher length of hospitalization presented more incidence by COVID-19 infection and mortality among those infected. We conclude that elective uro-oncological procedures at this epidemic period in a COVID-19-free institute are safe, and patients who need urgent procedures, with a long period of hospitalization need special care to avoid COVID-19 infection and its outcomes.
